# Attitudes toward evidence-based clinical practice among doctors of chiropractic with diplomate-level training in orthopedics

**DOI:** 10.1186/2045-709X-21-43

**Published:** 2013-12-06

**Authors:** Christopher B Roecker, Cynthia R Long, Robert D Vining, Dana J Lawrence

**Affiliations:** 1 Palmer College of Chiropractic, 1000 Brady Street, Davenport, IA 52803, USA; 2 Palmer Center for Chiropractic Research, 741 Brady Street, Davenport, IA 52803, USA

**Keywords:** Evidence based practice, Cross-sectional study, Chiropractic, Survey

## Abstract

**Background:**

Evidence-based clinical practice (EBCP) is a practice model gaining prominence within healthcare, including the chiropractic profession. The status of EBCP has been evaluated in a variety of healthcare disciplines, but little is known regarding the attitudes doctors of chiropractic (DCs) hold toward this model of healthcare. This project examines the attitudes toward EBCP within a specialty discipline of DCs.

**Methods:**

We identified a survey questionnaire previously used to evaluate EBCP among non-chiropractic complementary and alternative practitioners. We adapted this questionnaire for use among DCs and pretested it in 5 chiropractic college faculty. The final version was administered to DCs with diplomate-level training in orthopedics. The survey was emailed to 299 potential participants; descriptive results were calculated.

**Results:**

144 surveys were returned, resulting in a 48% response rate. The majority of respondents perceived EBCP as an important aspect of chiropractic practice. Respondents also believed themselves to have an above average skill level in EBCP, reported that training originated from their diplomate education, and based the majority of their practice on clinical research.

**Conclusion:**

Doctors of chiropractic with an orthopedic diplomate appear to have favorable attitudes toward EBCP. Further study will help understand EBCP perceptions among general field DCs. A logical next step includes validation of this questionnaire.

## Background

Evidence-based clinical practice (EBCP) is “the conscientious, explicit and judicious use of the best evidence when making decisions about the care of individual patients” [1]. This approach assists clinicians with clinical decision-making by integrating clinical expertise, patient values, and the best research evidence in an attempt to optimize clinical outcomes [1,2]. EBCP is a tool clinicians use to promote high-quality and consistent clinical care, justify clinical decision-making, and facilitate interdisciplinary cooperation [3-5]. This approach to clinical practice began formally in the early 1990s, has attracted widespread attention as an important method for improving patient outcomes, [6-8] and has become a foundational principle among healthcare professionals [9-15]. This transition to EBCP represents a dramatic advance within healthcare, which attempts to de-emphasize unsystematic clinical rationale and intuition in order to enhance clinical outcomes [6,16].

Efforts to promote EBCP within chiropractic have been identified as a fundamental component for advancing the profession [17-19]. Historically, doctors of chiropractic (DCs) have relied heavily on uncritical rationalism (deduction from theory) and uncritical empiricism (casual observation) as justification for chiropractic clinical practice [20,21]. More recently, however, the chiropractic profession is embracing the transition to EBCP, [22-24] though obstacles remain that can hinder acceptance of this mode of practice [25].

Even though adoption of EBCP by the chiropractic profession has been promoted by administrators and academics, [17] little is known regarding the attitudes practicing DCs hold toward EBCP. Assessments of EBCP have been conducted in many healthcare subspecialties, [26-34] but not within chiropractic. Investigating EBCP among practicing DCs is necessary to understand how EBCP is perceived and practiced, and to inform future research and educational opportunities.

Understanding the attitudes DCs have toward EBCP is an important waypoint for integrating EBCP into clinical practice. As a preliminary step, we surveyed EBCP perceptions in a specialty discipline of DCs. The objective of this project was to adapt a previously established EBCP questionnaire for use with DCs, pretest the questionnaire, and administer it to a convenience sample of DCs.

## Methods

DCs with postgraduate diplomate-level training in non-surgical chiropractic orthopedics (known as orthopedic diplomates) with membership in the Academy of Chiropractic Orthopedists (ACO) were surveyed in this study. Chiropractic orthopedic diplomates complete 300 hours of postgraduate education in non-surgical orthopedics and may represent the largest chiropractic group with diplomate-level training [35]. We conducted an anonymous, cross-sectional, online survey of EBCP perceptions held by members of the ACO. The Palmer College of Chiropractic Institutional Review Board approved this project.

### Survey questionnaire

A review of the literature demonstrated that the Evidence-Based Practice Attitudes and Utilization Survey (E-BASE) questionnaire [26] was an appropriate survey to adapt for specific application to DCs. This questionnaire has been validated [36] and successfully used to assess attitudes toward EBCP in a population of non-chiropractic complementary and alternative medicine (CAM) practitioners [26]. Permission to adapt the E-BASE survey for this project was provided by its author (Matthew J. Leach, RN, PhD, personal communication, Apr. 9, 2012).

Because the E-BASE survey was designed for use with a variety of non-chiropractic complementary and alternative medicine (CAM) practitioners, it required adaptation and pretesting prior to administration to a population of DCs. Adapting the E-BASE survey was primarily limited to modifying its terminology and design for use in an online format. The adapted questionnaire, specific to practicing DCs, is termed the Chiropractic Evidence-Based Practice Attitudes and Utilization Survey (Chiropractic E-BASE). The adapted questionnaire evaluates the following areas: the level of importance placed on EBCP; the level of skills respondents perceive they have to practice EBCP; the level of prior training to practice EBCP; EBCP practice perceptions; perceived barriers preventing the practice of EBCP; and perceived factors facilitating use of EBCP. It also collects anonymous demographic information.

### Pretesting

The adapted questionnaire was pretested on 5 chiropractic college faculty members with advanced training in EBCP to investigate content validity, clarity, and usability. After completing the online survey, the pretesters were interviewed and narrative feedback was collected.

This feedback informed us that the questionnaire took between 7 and 15 minutes to complete. Recommendations were limited to minor grammatical refinements to maximize clarity and usability within the online format. No suggestions were made to address content validity. All suggestions were incorporated into the Chiropractic E-BASE questionnaire.

### Eligibility criteria

This survey was performed with a convenience sample of DCs with active membership in the ACO who had provided an email contact to the ACO. Informed consent was required for participation and an online informed consent document was provided prior to initiating the online survey.

### Recruitment

The ACO Executive Board provided the email addresses to the ACO membership directory as of August 1, 2012. All ACO members with an active email address were invited to participate via email on August 1, 2012 and reminder emails were sent to non-responders at 1, 2, and 3 weeks after the initial invitation. Opt-out instructions were provided with each email contact.

### Study design

The online survey was administered by the online survey hosting site SurveyMonkey™ for the duration of 1 month (August 1, 2012 to August 31, 2012). Descriptive survey data were summarized in tables using frequency counts and percentages.

## Results

At the time of the survey, the ACO had 325 members, of which 309 (95%) had email addresses. Survey invitations were sent to all 309 email addresses, but 10 members had previously opted-out from all SurveyMonkey™ surveys. Therefore, 299 of the 325 active ACO members (92%) were available to participate. Of the 148 ACO members who then opened the link to the online survey, 144 agreed to the informed consent and proceeded to the Chiropractic E-BASE survey. The response rate for this project was 48% (144/299).

Demographics of the survey respondents are given in Table 1. The majority of respondents were male, older than 40 years of age, and had at least 16 years of practice experience. More than 40% were located in the Midwest United States and 58% practiced as a solo practitioner. Survey respondents were also likely to be members of the American Chiropractic Association (76%).

**Table 1:** **Characteristics of the Academy of Chiropractic Orthopedists responding to the chiropractic E-BASE (*n*** **= 129)**

**Variable**	**Category**	**Count**	**Percent (%)**
Age (years)			
	20-29	0	0
	30-39	11	9
	40-49	26	20
	50-59	63	49
	60-69	29	23
Sex			
	Male	117	91
Years since receiving Doctor of Chiropractic degree			
	<1	0	0
	1-5	2	2
	6-10	3	2
	11-15	13	10
	≥16	111	86
Years of chiropractic practice			
	<1	0	0
	1-5	1	1
	6-10	3	2
	11-15	13	10
	≥16	112	87
Predominant practice setting			
	Solo practice	75	58
	Group of CAM practitioners	26	20
	Combination of CAM and medical practitioners	10	8
	Institution (e.g. hospital or medical center)	10	8
	Educational institution	8	6

*CAM* complementary and alternative medicine.

Between 80-89% of the respondents agreed or strongly agreed that EBCP is necessary to chiropractic practice, improves the quality of patient care, assists them in making decisions about patient care, find research useful in their day-to-day practice, reported the prioritization of EBCP within chiropractic as a fundamental component for the future advancement of the profession, and are interested in improving their EBCP skills. Only 39% agreed or strongly agreed that EBCP takes into account a patient’s preference for treatment.

The majority of respondents believed they had above average skills when identifying answerable clinical questions and knowledge gaps, locating professional literature, and applying research evidence to patient care (Table 2).

**Table 2:** **The skill level chiropractic orthopedic diplomates have in evidence-based clinical practice (5-point Likert scale) (*n*** **= 143)**

	**1 (poor) (%)**	**2 (%)**	**3 (average) (%)**	**4 (%)**	**5 (advanced) (%)**
Identifying knowledge gaps in practice	0 (0)	4 (2.8)	32 (22.4)	75 (42.4)	65 (52.4)
Identifying answerable clinical questions	0 (0)	1 (0.7)	11 (7.7)	77 (53.8)	54 (37.8)
Locating professional literature	2 (1.4)	8 (5.6)	36 (25.2)	54 (37.8)	43 (30.1)
Online database searching	5 (3.5)	15 (10.5)	38 (26.6)	43 (30.1)	42 (29.4)
Retrieving evidence	5 (3.5)	16 (11.2)	43 (30.1)	46 (32.2)	33 (23.1)
Critical appraisal of the evidence	7 (4.9)	19 (13.3)	41 (28.7)	56 (39.2)	20 (14.0)
Applying research evidence to patient care	4 (2.8)	11 (7.7)	36 (25.2)	70 (49.0)	22 (15.4)
Using findings from clinical research	4 (2.8)	16 (11.2)	46 (32.2)	59 (41.3)	18 (12.6)
Using findings from systematic reviews	14 (9.8)	25 (17.5)	40 (28.0)	48 (33.6)	16 (11.2)
Synthesis of research evidence	8 (5.6)	18 (12.6)	58 (40.6)	45 (31.5)	14 (9.8)

With regard to where training in EBCP originated, 41% reported that it came from their diplomate education, while 15% reported it came via personal study, and a small percent (3%) noted it came during their chiropractic education. Additionally, 21% reported that applying research evidence to clinical practice was attributable to their diplomate education.

The majority (53%) reported that over half of their practice was based on clinical research evidence. Over two-thirds of respondents reported engaging in EBCP activities at least once within the past month, including reviewing clinical research (94%), online database searching (73%), and using professional literature to change clinical practice (88%) (Table 3). The sources of information used most frequently to inform clinical decision-making were traditional knowledge, published clinical evidence, and clinical practice guidelines (Table 4). The least common were trial and error and patient preference.

**Table 3:** **The extent evidence-based clinical practice within the past month among chiropractic orthopedic diplomates (*n*** **= 135)**

	**Never (%)**	**1-5 times (%)**	**6-10 times (%)**	**11-15 times (%)**	**≥16 times (%)**
I have read/reviewed professional literature related to my practice	2 (1.5)	64 (47.4)	26 (19.3)	12 (8.9)	31 (23.0)
I have read/reviewed clinical research findings related to my practice	8 (5.9)	72 (53.3)	22 (16.3)	11 (8.1)	22 (16.3)
I have used professional literature or research findings to assist my clinical decision-making	9 (6.7)	66 (48.9)	24 (17.8)	8 (5.9)	28 (20.7)
I have used professional literature or research findings to change my clinical practice	17 (12.6)	78 (57.8)	14 (10.4)	4 (3.0)	22 (16.3)
I have used an online database to search for practice related literature or research	37 (27.4)	53 (39.3)	19 (14.1)	7 (5.2)	19 (14.1)
I have used and online search engine to search for practice related literature or research	8 (5.9)	48 (35.6)	37 (27.4)	16 (11.9)	26 (19.3)
I have consulted a colleague or industry expert to assist my clinical decision-making	24 (17.8)	77 (57.0)	14 (10.4)	6 (4.4)	14 (10.4)
I have referred to magazines, layperson/self-help books, or non-government/non-education institution websites to assist my clinical decision-making	61 (45.2)	51 (37.8)	12 (8.9)	4 (3.0)	7 (5.2)

**Table 4:** Sources of information chiropractic orthopedic diplomates use to inform clinical decision-making^* ^**(*n*** **= 135)**

	**Average ranking (SD)**
Traditional knowledge	3.20 (2.46)
Published clinical evidence (e.g. clinical trials)	3.67 (2.56)
Clinical practice guidelines	4.55 (2.73)
Textbooks	5.01 (2.55)
Consulting fellow practitioners or experts	5.47 (2.01)
Personal intuition	5.56 (2.60)
Personal preference	6.53 (2.67)
Published experimental/laboratory evidence (e.g. animal or test tube studies)	6.64 (2.96)
Patient preference	6.98 (2.31)
Trial and error	7.41 (2.53)

^*^Sources were ranked as most-to-least frequently used (1–10), respectively.

More than 80% of respondents perceived that the barriers to implementing EBCP included a lack of clinical evidence in CAM and a lack of time (Table 5). Issues reported to not act as a barrier to EBCP were lack of resources (65%), lack of interest in EBCP (55%), lack of relevance to chiropractic practice (48%), and lack of colleague support (47%) (Table 5). Factors reported to be moderately to very useful for facilitating EBCP included access to the internet at work (96%), free online databases at work (70%), online EBCP educational material (89%), critical reviews of research within the chiropractic profession (90%), and the ability to download full-text articles (82%).

**Table 5:** **Barriers preventing chiropractic orthopedic diplomates from practicing evidence-based clinical practice (*n*** **= 132)**

	**Not a barrier (%)**	**A minor barrier (%)**	**A moderate barrier (%)**	**A major barrier (%)**
Lack of clinical evidence in CAM	18 (13.6)	42 (31.8)	58 (43.9)	14 (10.6)
Lack of industry support for EBCP	50 (37.9)	58 (43.9)	22 (16.7)	2 (1.5)
Lack of time	18 (13.6)	48 (36.4)	43 (32.6)	23 (17.4)
Insufficient skills for locating research	50 (37.9)	54 (40.9)	21 (15.9)	7 (5.3)
Insufficient skills for interpreting research	45 (34.1)	55 (41.7)	24 (18.2)	8 (6.1)
Insufficient skills to critically appraise/evaluate the literature	40 (30.3)	60 (45.5)	25 (18.9)	7 (5.3)
Insufficient skills to apply research findings to clinical practice	44 (33.3)	65 (49.2)	20 (15.2)	3 (2.3)
Patient preference for treatment	50 (37.9)	58 (43.9)	22 (16.7)	2 (1.5)
Lack of resources	86 (65.2)	33 (25.0)	12 (9.1)	1 (0.8)
Lack of incentive to participate in EBCP	48 (36.4)	41 (31.1)	29 (22.0)	14 (10.6)
Lack of interest in EBCP	73 (55.3)	45 (34.1)	7 (5.3)	7 (5.3)
Lack of relevance to chiropractic practice	63 (47.7)	47 (35.6)	19 (14.4)	3 (2.3)
Lack of colleague support for EBCP	62 (47.0)	38 (28.8)	24 (18.2)	8 (6.1)

*CAM* complementary and alternative medicine.

*EBCP* evidence-based clinical practice.

## Discussion

To our knowledge this study represents the first survey of practicing DCs directly related to EBCP. We found that participants held favorable attitudes toward EBCP and consider this model to be an important component for the current practice and advancement of the chiropractic profession. The positive perceptions reported in this survey are comparable to the positive self-reported perceptions of other healthcare professionals [26-29,31-34]. Our results suggest that the majority of DCs with diplomate-level training in orthopedics embrace EBCP while serving as the first measure of EBCP perceptions within a subset of practicing DCs.

Respondents reported the majority of their EBCP knowledge originated from the postgraduate orthopedic diplomate training program. While postgraduate training in chiropractic orthopedics may incorporate aspects of EBCP, these competencies are not officially part of the curriculum (ACO Executive Board, personal communication, July 2, 2013). Despite this, it is evident that chiropractic orthopedic diplomates perceive their postgraduate educational program as a source of EBCP training. It is likely this originates from the informal inclusion of EBCP concepts into the training program.

This survey found that the majority of respondents were interested in having access to EBCP educational material. These findings are consistent with the results of other surveys intended to highlight factors facilitating EBCP in other healthcare disciplines [26,29,34]. Current continuing chiropractic education opportunities focused on the principles of EBCP are uncommon and may represent an unmet subject area. Also, because the Council on Chiropractic Education has recently established EBCP competency requirements for U.S. chiropractic education, [24] providing postgraduate EBCP educational opportunities for DCs trained prior to the implementation of these educational competencies is important. Future efforts should be directed toward developing focused postgraduate EBCP educational opportunities for DCs.

The information sources used to inform clinical decision-making reported in this survey were nearly identical to those used by other CAM practitioners [26]. These findings prioritized clinical information obtained from traditional knowledge, published clinical evidence, and clinical practice guidelines as the most frequently used source of clinical information. Interestingly, patient preference was rated as one of the least frequently used sources of information. Because the primary objective of EBCP is to integrate the clinician’s clinical expertise, the patient’s values, and the best research evidence, [16] these results may indicate respondents de-emphasize incorporating the patient’s preference into clinical decision-making. Traditional knowledge was also reported as the most common source of information for clinical decision-making. Unfortunately, traditional knowledge was not further defined and we are uncertain how each respondent interpreted this source of information. Whether respondents assumed traditional knowledge to indicate intuitive ways of knowing or knowledge resulting from clinical experience has a substantial impact on the interpretation of this finding. Further refinement of this response identifies an area of improvement for future evaluations of the Chiropractic E-BASE questionnaire. A persistent criticism of EBCP is that it neglects aspects of clinical decision-making not resulting from clinical research, [15] even though the core concept of this model is to incorporate the clinical expertise, patient preference, and the best available evidence when making clinical decisions [16]. Whether DCs perceive clinical decision-making to originate from the amalgamation of clinical experience, patient preferences, and the best available research evidence is another area of improvement for future refinement of the Chiropractic E-BASE questionnaire.

It is imperative that the results of this survey be considered in the context of self-rated perceptions. It has been argued that the accuracy of self-reporting is poor [37] and result in over-estimating competence of actual EBCP performance and knowledge [27,38,39]. Future investigations into whether responses to the Chiropractic E-BASE questionnaire are associated with actual performance are warranted. It is also important to assess the EBCP perceptions of a broader sample of DCs.

### Study limitations

This project has 3 important limitations. First, while every attempt was made to maximize the response rate, we are unable to assess the generalizability of our sample to the total population of chiropractic orthopedic diplomates. Our sample was a convenience sample of ACO members limited to those with email addresses who did not previously opt-out from SurveyMonkey™ surveys.

Second, the pretesting phase of the Chiropractic E-BASE questionnaire for this project was not a substitution for formal validation techniques. Validation of the Chiropractic E-BASE questionnaire is an important next step in this line of inquiry. Therefore, the results of this survey are descriptive and are intended to inform future development of the Chiropractic E-BASE questionnaire.

Lastly, the number of respondents decreased as the survey progressed through each section, presumably from dropout. There were 15 respondents who failed to complete the survey, which corresponds to 10% of all respondents. Respondents who failed to complete to survey were not contacted to investigate the reason for dropout.

## Conclusion

This study is a first step in investigating the perceptions, knowledge and use of EBCP within DCs. Chiropractic orthopedic diplomates perceive EBCP to be important to the practice of chiropractic and fundamental to the advancement of the profession. Access to resources was reported to facilitate the use of EBCP and an emphasis on EBCP continuing education may allow more DCs to become familiar with EBCP. Further refinement of the Chiropractic E-BASE questionnaire is needed to investigate whether clinical decision-making is informed by the combination of clinical expertise, patient preference, and best available research evidence.

## Abbreviations

EBCP: Evidence-based clinical practice; ACO: Academy of Chiropractic Orthopedists; CAM: Complementary and alternative medicine; E-BASE: Evidence-Based Practice Attitudes and Utilization Survey.

## Competing interests

The authors declare that they have no competing interests.

## Authors’ contributions

Each of the authors contributed to the conception and methods development of this project. CBR carried out the adaptation of the Chiropractic E-BASE questionnaire, created the online survey format, administered the pretesting, sent survey requests to the ACO membership, and drafted the initial manuscript. CRL, RDV, and DJL provided substantive intellectual contributions and assisted in revising the manuscript into final form. All authors approved the final manuscript.

## Authors’ information

CBR is an Instructor within the Life Sciences Department at Palmer College of Chiropractic, Davenport, IA.

CRL is a Professor and Director of Research at Palmer College of Chiropractic, Davenport IA.

RDV is an Assistant Professor at the Palmer Center for Chiropractic Research, Palmer College of Chiropractic, Davenport, IA.

DJL is the Senior Director for the Center for Teaching and learning at Palmer College of Chiropractic, Davenport, IA.
